# Impact of glycemic control on aortic stiffness, left ventricular mass and diastolic longitudinal function in type 2 diabetes mellitus

**DOI:** 10.1186/s12933-017-0557-z

**Published:** 2017-06-17

**Authors:** Michaela Kozakova, Carmela Morizzo, Alan G. Fraser, Carlo Palombo

**Affiliations:** 1 Department of Clinical and Experimental Medicine, University of Pisa, Pisa, Italy; 2 Esaote SpA, Genoa, Italy; 3 School of Medicine, Department of Surgical, Medical and Molecular Pathology and Critical Care Medicine, University of Pisa, Via Savi 10, 56126 Pisa, Italy; 4 Department of Cardiology, University Hospital of Wales, Heath Park, Cardiff, CF14 4XW UK

**Keywords:** Glycemic control, LV diastolic function, Aortic stiffness, LV mass, Arterio-ventricular coupling, Systo-diastolic coupling

## Abstract

**Background:**

Poor glycemic control is associated with impaired left ventricular (LV) diastolic function in patients with type 2 diabetes mellitus (T2DM). Inappropriate LV mass increase and accelerated aortic stiffening were suggested to participate on deterioration of diastolic function. The present study investigated the inter-relationships between glycemic control, early diastolic and systolic longitudinal velocity of mitral annulus, LV mass and aortic stiffness in T2DM patients free of cardiovascular disease and with preserved LV ejection fraction, and compared them with those observed in healthy volunteers of similar age and sex distribution.

**Methods:**

125 T2DM patients and 101 healthy volunteers underwent noninvasive measurement of systolic (s′) and early diastolic (e′) velocities of mitral annulus, LV mass, carotid-femoral pulse wave velocity (cfPWV) and local carotid blood pressure (BP).

**Results:**

Forty-four (35.2%) T2DM patients had e′ velocity lower than that expected for age (against 7.9% in healthy volunteers; *P* < 0.0001), 34 (27.2%) had cfPWV higher than that expected for age and mean BP (against 5.9% in healthy volunteers; *P* < 0.0001), and 71 (56.8%) had LV mass higher than that expected for body size and stroke work (against 17.6% in healthy volunteers; *P* < 0.0001). Carotid systolic BP was higher in T2DM patients (124 ± 14 vs 111 ± 11 mmHg; *P* < 0.0001). In multivariate analysis, e′ velocity was independently related to age, carotid BP and s′ velocity in healthy volunteers, and to male sex, age, carotid BP, heart rate and LV mass in T2DM. Glycosylated hemoglobin (HbA1c) was independently related to cfPWV and LV mass in T2DM patients. T2DM patients with HbA1c ≥6.5% (N = 85) had higher cfPWV (*P* < 0.05), central BP (*P* = 0.01), prevalence of LV hypertrophy (*P* = 0.01) and lower e′ and s′ velocity (*P* = 0.001 and <0.05, respectively) as compared to those with HbA1c <6.5%.

**Conclusions:**

One-third of T2DM patients with preserved LV ejection fraction has sign of subclinical LV diastolic dysfunction. HbA1c levels are positively associated with LV mass and aortic stiffness, both of which show a negative independent impact on early diastolic velocity e′, the latter through an increase in afterload. T2DM patients with suboptimal glycemic control (HbA1c ≥ 6.5%) have lower diastolic and systolic LV longitudinal performance, together with increased aortic stiffness and a higher prevalence of LV hypertrophy.

## Background

Left ventricular (LV) diastolic dysfunction is considered an early manifestation of diabetic heart disease [1–3], and type 2 diabetes mellitus (T2DM) and hyperglycemia play an important role in the development and prognosis of diastolic heart failure, i.e. heart failure with preserved ejection fraction [4, 5]. Poor glycemic control may affect diastolic function by several mechanisms [6]. Chronic increase in plasma glucose levels elicits increase in LV mass through cardiomyocyte hypertrophy, collagen deposition and cross-linking [7–9]. Hyperglycemia also damages mitochondrial energy signaling, inhibits autophagic flux in hypertrophic cardiomyocytes [10] and enhances myocardial oxidative stress [11], all of which may result in a deterioration of LV diastolic performance. Finally, hyperglycemia induces collagen cross-linking in arterial wall, thus accelerating large artery stiffening [12]. Increase in large artery stiffness and consequent increase in arterial load, above all in late-systolic load, affects the transition from myocardial contraction to relaxation, causing a slower rate of diastolic LV pressure fall [13, 14]. Increased vascular load and wall stress stimulates LV hypertrophy and remodeling that can further deteriorate myocardial function.

LV diastolic function is determined by relaxation rate, restoring forces and passive compliance of LV wall. There is no single noninvasive measure that can describe all these mechanisms, yet the LV lengthening velocity e′, measured by tissue Doppler imaging, represents a valuable parameters of diastolic function as it reflects both relaxation and restoring forces [15, 16]. In addition, e′ velocity has been shown to be inversely related to serum concentration of procollagen type I carboxy-terminal propeptide [17], which is considered a marker of myocardial collagen content [18]. Previous studies have reported reduced e′ velocity of mitral annulus in asymptomatic T2DM patients [19, 20] as well as an association between e′ velocity and poor glycemic control [19, 21]. They have also demonstrated an inverse relationship between e′ velocity and LV mass or arterial stiffness [22, 23] and a direct association between diastolic e′ and systolic s′ longitudinal velocities [23]. Yet, the mutual relationships between glycemic control, LV hypertrophy, arterial stiffening, afterload, systolic performance and LV diastolic function were not clearly established in diabetic patients.

Therefore, the present cross-sectional study evaluated the inter-relationships between LV diastolic longitudinal velocity e′ and LV mass, aortic and carotid stiffness, central blood pressure (BP), systolic velocity s′ and indicators of glycemic exposure (plasma levels of fasting glucose and glycosylated hemoglobin and duration of diabetes) in T2DM patients free of clinical cardiovascular disease and with preserved LV ejection fraction (EF). Observed associations were compared with those obtained in a group of healthy volunteers of comparable age and sex distribution.

## Methods

### Study population

We investigated two populations. The first population consisted of 125 T2DM patients (aged between 40 and 71 years) free of clinical cardiovascular diseases, with preserved LV EF, without signs of diastolic heart failure and with normal glomerular filtration rate (≥60 mL/min/1.73 m^2^). They were selected from those referred for a complete vascular and cardiac examination to the Clinic for Cardiometabolic Risk Prevention of the Department of Surgical and Medical Pathology, University of Pisa. The second population consisted of 101 apparently healthy volunteers, comparable for age and sex distribution, selected from those participating on the study Relationship between Insulin Sensitivity and Cardiovascular Risk (RISC) [24]. In both groups, cardiovascular disease was excluded by clinical history, resting ECG and echocardiography; on echocardiographic examination LV ejection fraction was ≥52 and 54%, for men and women, respectively [25]. LV asynergy was absent and E/e′ ratio (index of filling pressure) was <13.0 [26]. Hypertension was defined as either systolic BP ≥140 mmHg or diastolic BP ≥90 mmHg at two study visits, or current antihypertensive treatment. Antagonists of renin-angiotensin-aldosterone system (ACE inhibitors and ARBs) were the most frequently used anti-hypertensive agent, followed by beta-blockers. Diagnosis of T2DM was based on plasma glucose criteria (fasting glucose ≥7 mmol/L or HbA1c ≥6.5% or 2-h glucose during oral-glucose tolerance test ≥11.1 mmol/L) [27]. Twenty-two T2DM patients were treated by diet, 94 by oral antidiabetic drugs, 7 by a combination of oral antidiabetics and insulin and 2 by insulin only.

### Study protocol

The protocol of the study followed the principles of the Declaration of Helsinki and was approved by the institutional ethics committee (reference number: 3146/2010 and 245/2015). All subjects gave their informed consent to participate.

#### Vascular examination

All study subjects underwent carotid ultrasound and measurement of carotid-femoral pulse wave velocity (cfPWV). Vascular examination was performed in the afternoon, 3 h after a light meal, in a quiet room with a stable temperature of 22°, after resting comfortably for at least 15 min in the supine position. All subjects were asked to abstain from cigarette smoking, caffeine and alcohol consumption and vigorous physical activity for 24 h.

Carotid-femoral pulse wave velocity (cfPWV) was measured according to current guidelines using the Complior device (Alam Medical, Vincennes, France). Regression equations obtained in a reference value population were used to calculate appropriate cfPWV value according to age and mean BP for each study subjects [28]. In our laboratory, intra- and inter-individual variability of cfPWV measurement are 4.3 ± 2.8 and 5.1 ± 2.9%, respectively.

Carotid ultrasound was performed by a single operator (CM) on the right common carotid artery using an ultrasound scanner equipped with a 10 MHz linear probe (MyLab 70, Esaote, Genova, Italy) and implemented with a previously validated radiofrequency-based tracking of arterial wall that allows a real-time determination of common carotid distension (QAS^®^) with high spatial and temporal resolution (sampling rate of 550 Hz on 32 lines) [29]. From the distension curves, one-point carotid PWV (ccaPWV) was calculated applying the Bramwell-Hill equation that relates the propagation velocity to arterial distensibility [30]. The local carotid pressure was estimated by converting the distension curve to a pressure curve using a linear conversion factor and assuming that the difference between mean arterial pressure and diastolic pressure is invariant along the arterial tree [31]. The peripheral BP needed for rescaling carotid waveforms was measured at the left brachial artery (Omron, Kyoto, Japan) during each acquisition of the distension curves. Local carotid pressure estimate by QAS was previously validated against applanation tonometry [29] and was used as a surrogate of central aortic pressure.

All radiofrequency-derived measures were averaged over 6 consecutive cardiac beats and the values used for statistical analysis represent a mean of three consecutive acquisitions. In our laboratory intra-individual variability of common carotid artery distension by QAS was 7.5 ± 4.6% [29].

#### Cardiac ultrasound

Cardiac ultrasound was performed by a single operator (CM) with a standard ultrasound system (MyLab 70, Esaote, Genova, Italy) equipped with a 3.5-MHz, phased-array probe. LV EF was measured by biplane method of disks [25]. LV dimensions and wall thickness were measured in M-mode images and LV mass was calculated as recommended [32]. LV hypertrophy was considered when LV mass indexed for height^2.7^ was >49 g/m^2.7^ in men and >45 g/m^2.7^ in women [25]. The ratio of observed LV mass and LV mass predicted for gender, body height and stroke work was calculated, and inappropriate increase in LV mass was considered when the ratio was >1.28 [33]. End-systolic stress corrected midwall shortening (ess-MWS) was calculated as described [34]. Stroke volume was assessed as the product of aortic valve cross-sectional area and transaortic flow-velocity time integral. Transmitral flow pattern was obtained and velocity of early (E) and late (A) diastolic filling was measured and E/A ratio was calculated.

LV longitudinal velocities at mitral annular level, both at septal and lateral sides, were measured by color-guided pulsed-wave tissue Doppler in the apical four-chamber view. The sample volume was placed at the junction of the LV wall with the mitral annulus, and the cursor was aligned so that the angle of incidence between the Doppler beam and the longitudinal motion of the LV was as close as possible to 0° [35]. From spectral traces, peak systolic longitudinal velocity (s′) and peak longitudinal velocity during early diastolic filling (e′) were measured and averaged over five consecutive cardiac cycles. Reported values of s′ and e′ longitudinal velocities represent an average of septal and lateral sides. The ratio between transmitral E and e′ velocity (E/e′ ratio) was calculated [26]. The intra-individual variability of tissue Doppler measurements in our laboratory is 5.8 ± 4.3 and 6.3 ± 4.8% for s′ and e′ velocity, respectively.

Since e′ velocity depends strongly on age, a linear regression equation describing the relationship between age and e′ velocity in 101 healthy volunteers was applied to calculate the age-related normal e′ value and the respective lower 95% tolerance interval in each study subject. If the deficit of observed e′ value to the age-related normal e′ value was greater than the lower 95% tolerance interval, the subject was considered having diastolic dysfunction [20].

#### Medical history and physical examination

A standardized medical history, physical examination and resting ECG were performed in all subjects. Height and weight were obtained, and body mass index (BMI) was calculated as body weight (in kg) divided by squared height (in meters). Waist circumference was measured as the narrowest circumference between the lower rib margin and anterior superior iliac crest. Office brachial BP was measured twice during two different visits in a seated patient, using a standard mercury sphygmomanometer; regular or large adult cuffs were used, depending on patient arm circumference. The mean value of the two measurements was calculated and used for statistical analysis.

#### Analytical procedures

All biochemical parameters (LDL-cholesterol, HDL-cholesterol, triglycerides, glucose) were determined within 1 week of cardiovascular examination by standard methods on a Roche-Modular Autoanalyzer (Milan, Italy). Glycosylated hemoglobin (HbA1c) was measured by high-performance liquid chromatography and standardized against DCCT standard.

#### Statistical analysis

Data are expressed as mean ± SD, categorical data as percentages. Variables with skewed distribution (triglycerides, HbA1c) were summarized as median [interquartile range], and were logarithmically transformed for parametric statistical analysis. ANOVA was used to compare continuous variables, and a χ^2^ test for categorical variables. The univariate relationships between the outcome variables and continuous variables were assessed by Pearson’s correlation coefficient. Multiple linear regression analyses (controlled for sex, current smoking and, in T2DM patients, also for BP-lowering, lipid-lowering and anti-diabetic treatment) with backward stepwise removal were used to identify the independent associations of outcome variables with their significant univariate correlates. Statistical tests were two-sided, and significance was set at a value of *P* < 0.05. Statistical analysis was performed by JMP software, version 3.1 (SAS Institute Inc., Cary, NC, USA).

## Results

### Characteristics of the study populations

The two populations were analyzed separately and their main characteristics are reported in Table 1. T2DM patients and healthy volunteers had comparable age and sex distribution. T2DM patients had higher BMI, waist circumference, peripheral BP and heart rate, higher triglycerides and plasma glucose levels and lower HDL-cholesterol. Within T2DM patients 66.4% had hypertension, 52.8% were treated by BP-lowering therapy and 44.0% by lipid-lowering therapy. Table 2 reports cardiac and vascular measures in both groups; T2DM patients had higher LV mass, LV mass index, prevalence of LVH and inappropriate LV mass and decreased ess-MWS as well as longitudinal e′ and s′ velocities. T2DM patients had also higher cfPWV, ccaPWV and local carotid BP. Thirty-four T2DM patients (27.2%) and 6 healthy volunteers (5.9%; *P* < 0.0001) had cfPWV higher than that expected for age and mean BP [28].

**Table 1:** Characteristics of study populations

	T2DM patients	Healthy volunteers	*P*
	Mean ± SD/median [IQR]	Mean ± SD/median [IQR]	
Gender (M:F)	82:43	61:40	0.45
Age (years)	58 ± 7	57 ± 8	0.24
Height (cm)	170 ± 9	171 ± 9	0.36
BMI (kg/m^2^)	28.6 ± 4.2	26.1 ± 3.6	<0.0001
Obesity (%)	26.4	9.0	0.0001
Waist circumference (cm)	104 ± 11	93 ± 10	<0.0001
Heart rate (bpm)	68 ± 11	62 ± 10	<0.0001
Systolic/diastolic BP (mmHg)	138 ± 17/80±9	121 ± 11/75±7	<0.0001
LDL-cholesterol (mmol/L)	2.89 ± 0.72	3.11 ± 0.93	0.10
HDL-cholesterol (mmol/L)	1.28 ± 0.31	1.48 ± 0.34	<0.0005
Triglycerides (mmol/L)	1.33 [0.94]	1.11 [0.73]	0.001
Fasting glucose (mmol/L)	7.0 ± 1.8	5.1 ± 0.5	<0.0001
HbAc1 (%)	7.0 [1.3]		
T2DM duration (years)	7.1 ± 6.4		
Current smoking (%)	35.2	29.1	0.26
BP-lowering treatment (%)	52.8	0	<0.0001
Lipid-lowering treatment (%)	44.0	0	<0.0001

**Table 2:** Cardiovascular parameters in study populations

	T2DM patients	Healthy volunteers	*P*
	Mean ± SD	Mean ± SD	
LV mass (g)	207 ± 41	176 ± 42	<0.005
LV mass index (g/m^2.7^)	50 ± 11	41 ± 9	<0.0001
LV hypertrophy (%)	51.2	18.6	<0.0001
Inappropriate LV mass (%)	56.8	17.6	<0.0001
Ejection fraction (%)	65 ± 7	66 ± 8	0.29
ess-MWS (%)	101 ± 15	108 ± 11	<0.005
Stroke volume (mL)	72 ± 17	76 ± 17	0.15
E/A ratio transmitral	0.90 ± 0.24	1.10 ± 0.27	<0.0001
E/e′ ratio	7.4 ± 2.1	5.9 ± 1.4	<0.0001
e′ velocity (cm/s)	9.4 ± 1.7	11.5 ± 2.2	<0.0001
s′ velocity (cm/s)	9.1 ± 1.4	9.6 ± 1.4	<0.05
cfPWV (m/s)	8.5 ± 1.8	7.1 ± 1.3	<0.0001
ccaPWV (m/s)	8.4 ± 1.8	7.1 ± 1.6	<0.0005
Carotid systolic BP (mmHg)	124 ± 14	111 ± 11	<0.0001
Carotid pulse pressure (mmHg)	44 ± 13	35 ± 9	<0.0001

### Univariate correlations

Table 3 reports univariate correlations of e′ velocity, cfPWV and LV mass with age, body size, hemodynamic and metabolic variables (reported r values with *P* at least <0.05), separately for T2DM patients and healthy volunteers. In healthy volunteers, ccaPWV correlated with age, heart rate, systolic BP, LV mass and cfPWV (r = 0.52, 0.21, 0.54, 0.21 and 0.43; *P* < 0.05–0.0001), and in T2DM patients ccaPWV correlated with waist circumference, BMI, heart rate, systolic BP and cfPWV (r = 0.22, 0.18, 0.25, 0.38 and 0.21; *P* < 0.05–0.0001).

**Table 3:** Univariate correlation coefficients describing the associations of early diastolic velocity, cfPWV and LV mass with tested parameters in T2DM patients and in healthy volunteers

	T2DM patients			Healthy volunteers		
	e′ (cm/s)	cfPWV (m/s)	LV mass (g)	e′ (cm/s)	cfPWV (m/s)	LV mass (g)
cfPWV (m/s)	−0.32	–	0.21	−0.47	–	0.26
Carotid systolic BP (mmHg)	−0.38	0.44	0.22	−0.58	0.46	0.36
Carotid pulse pressure (mmHg)	−0.31	0.26	ns	−0.40	0.44	0.29
Age (years)	−0.31	0.29	ns	−0.61	0.47	0.35
BMI (kg/m^2^)	ns	0.21	0.25	ns	ns	0.37
Waist circumference (cm)	ns	0.23	0.45	−0.24	0.25	0.45
Heart rate (bpm)	−0.25	0.24	0.20	ns	0.22	ns
Stroke volume (mL)	0.19	ns	ns	ns	ns	0.43
LV mass (g)	−0.31	0.21	–	−0.32	0.26	–
Log triglycerides	ns	ns	ns	ns	ns	ns
LDL-cholesterol (mmol/L)	ns	ns	ns	ns	ns	ns
HDL-cholesterol (mmol/L)	ns	ns	ns	ns	ns	−0.27
Fasting glucose (mmol/L)	−0.23	0.18	0.24	−0.29	0.32	ns
Log HbA1c	−0.27	0.37	0.39	–	–	–
T2DM duration (years)	ns	0.21	0.34	–	–	–

r value reported when *P* at least < 0.05

In healthy volunteers, the inverse linear relationship between e′ velocity and age (r = −0.60; *P* < 0.0001) was described by the equation e′ velocity (cm/s) = −0.168 * Age (years) + 21.03, and by the lower 95% tolerance interval of −2.47 cm/s. Applying this equation and the lower 95% tolerance interval as previously described [20], we identified 44 T2DM patients (35.2%) and 8 healthy volunteers (7.9%; *P* < 0.0001) having diastolic dysfunction.

**Fig. 1:**
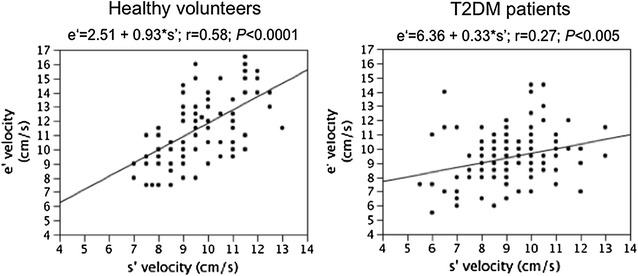
Relationship between s′ and e′ longitudinal velocities in Healthy volunteers and in T2DM patients

A correlation between e′ and s′ velocity was observed both in healthy volunteers and in T2DM patients (Fig. 1); the relationship was stronger and the slope of the regression line was steeper (*P* < 0.0001) in healthy subjects than in T2DM patients.

### Independent determinants of e′ velocity, cfPWV, LV mass and ccaPWV

The independence of the associations of e′ velocity, cfPWV, LV mass and ccaPWV with their univariate correlates was tested in multiple regression models with stepwise removal, adjusted for sex and smoking habit and, in T2DM patients, also for BP-lowering, lipid-lowering and anti-diabetic treatment (Table 4). Early diastolic longitudinal velocity e′ in healthy volunteers was determined by age, carotid systolic BP and s′ velocity; in T2DM patients it was determined by male sex, age, carotid systolic BP, heart rate and LV mass. CfPWV was independently associated with age, carotid BP, heart rate and fasting glucose in healthy volunteers, and with age, carotid BP, heart rate and HbA1c levels in T2DM patients. LV mass was determined by age, body height, carotid systolic BP and stroke volume in healthy volunteers, and by male sex, waist circumference, carotid systolic BP, HbA1c levels and diabetes duration in T2DM patients. CcaPWV was determined by age, systolic BP and heart rate in healthy volunteers (ß ± SE: 0.34 ± 0.09, 0.34 ± 0.09 and 0.17 ± 0.07; cumulative *R* ^*2*^ = 0.40; *P* < 0.0001), and by heart rate, systolic BP and hypertensive treatment in T2DM patients (ß ± SE: 0.21 ± 0.08, 0.32 ± 0.08 and 0.18 ± 0.08; cumulative *R* ^*2*^ = 0.21; *P* < 0.0001).

**Table 4:** Independent determinants (β ± SE) of early diastolic velocity, cfPWV and LV mass in T2DM patients and in healthy volunteers

	T2DM patients			Healthy volunteers		
	e′ velocity (cm/s)	cfPWV (m/s)	LV mass (g)	e′ velocity (cm/s)	cfPWV (m/s)	LV mass (g)
Sex (male)	0.19 ± 0.08		0.18 ± 0.08			
Age (years)	−0.22 ± 0.08	0.22 ± 0.07		−0.37 ± 0.08	0.34 ± 0.10	0.25 ± 0.08
Height (cm)						0.45 ± 0.07
Waist circumference (cm)			0.26 ± 0.07			
Carotid SBP (mmHg)	−0.25 ± 0.08	0.28 ± 0.07	0.18±0.07	−0.24 ± 0.08	0.26 ± 0.09	0.28 ± 0.08
Heart rate (bpm)	−0.24 ± 0.08	0.18 ± 0.07			0.30 ± 0.08	
Stroke volume (mL)						0.25 ± 0.07
LV mass (g)	−0.25 ± 0.09					
s′ velocity (cm/s)				0.43 ± 0.06		
Fasting glucose (mmol/L)					0.29 ± 0.08	
Log HbA1c		0.25 ± 0.07	0.19 ± 0.08			
T2DM duration (years)			0.22 ± 0.07			
Cumulative R^2^	0.32 *P* < 0.0001	0.34 *P* < 0.0001	0.38 *P* < 0.0001	0.60 *P* < 0.0001	0.43 *P* < 0.0001	0.56 *P* < 0.0001

*SBP* systolic BP

### Poor glycemic control and cardiovascular measures

T2DM patients were divided according to HbA1c levels (<6.5% and ≥6.5%) [27]. Table 5 demonstrates that the two subgroups were comparable for age, sex and T2DM duration. As compared to patients with HbA1c <6.5%, patients with HbA1c ≥6.5% had higher carotid systolic BP, cfPWV and LVMI, higher prevalence of diastolic dysfunction, increased aortic stiffness and LVH, lower e′ and s′ longitudinal velocities and ess-MWS. No differences between the two subgroups were observed for ccaPWV.

**Table 5:** Cardiovascular measures in T2DM patients according to HbA1c levels

	HbA1c <6.5%	HbA1c ≥6.5%	*P*
N	40	85	
Age (years)	58 ± 8	58 ± 10	0.74
Male:female	25:15	60:25	0.37
T2DM duration (years)	6.0 ± 5.2	7.6 ± 6.8	0.18
HbAc1 (%)	6.1 ± 0.3	7.6 ± 0.9	<0.0001
Carotid systolic BP (mmHg)	119 ± 13	126 ± 14	0.01
cfPWV (m/s)	8.0 ± 1.5	8.7 ± 1.9	<0.05
Increased cfPWV (%)	15.0	32.9	<0.05
ccaPWV (m/s)	8.3 ± 1.6	8.5 ± 1.8	0.31
LVMI (g/m^2.7^)	46 ± 10	52 ± 10	0.005
LVH (%)	35.0	58.8	0.01
e′ velocity (cm/s)	10.1 ± 1.6	9.1 ± 1.7	0.001
Diastolic dysfunction (%)	22.5	40.0	<0.05
s′ velocity (cm/s)	9.5 ± 1.5	8.9 ± 1.4	<0.05
ess-MWS (%)	105 ± 13	99 ± 16	<0.05

## Discussion

In T2DM patients free of cardiovascular disease and with preserved LV EF, HbA1c levels were positively associated with LV mass and aortic stiffness, both of which showed a negative independent impact on early diastolic velocity e′, the latter through an increase in central systolic BP. T2DM patients with suboptimal glycemic control (HbA1c ≥ 6.5%) had a higher prevalence of LV diastolic dysfunction, together with a higher prevalence of LV hypertrophy and increased aortic stiffness.

LV diastolic dysfunction is an early manifestation of diabetic heart disease [1, 20, 21], and a subclinical impairment of diastolic function is associated with higher glucose levels [3, 19, 21]. Chronic increase in plasma glucose levels has been shown to negatively influence LV diastolic performance through different mechanisms, including alteration in mitochondrial energy metabolism and increment in myocardial oxidative stress [10, 11], LV mass increase and changes in myocardial composition [7, 8], acceleration of large artery stiffening and consequent increase in vascular load [12–14]. The present study was designed to define the interplay between glycemic control, arterial stiffening, LV hypertrophy and subclinical diastolic dysfunction in asymptomatic T2DM patients with preserved LV ejection fraction.

### Prevalence of impaired LV diastolic function

E′ velocity of mitral annulus is considered a valuable noninvasive parameter of LV diastolic function, as it reflects myocardial relaxation and restoring forces [15, 16] as well as myocardial fibrosis [17, 18]. Yet, e′ velocity depends strongly on age; with age the magnitude of e′ velocity progressively decreases. In order to determine the age-corrected values for normal e′ velocity, which can be used to identify T2DM patients with impaired diastolic function, we utilized a previously described method [20] employing a linear regression equation describing the relationship between e′ velocity and age in healthy volunteers of comparable age and sex distribution. Applying this approach, one-third of asymptomatic T2DM patients with preserved ejection fraction had e′ velocity lower than that expected for age.

### HbA1c levels, LV mass, aortic stiffness and e′ velocity

Fasting plasma glucose and HbA1c levels were not independently related to e′ velocity, yet HbA1c was directly and independently associated with LV mass and aortic stiffness, as assessed by cfPWV. The association between glycemic control and LV mass has been previously described in both non-diabetic and diabetic patients [36, 37], and it is supposed to reflect a glucose-induced activation of epigenetic mechanism regulating cardiomyocyte hypertrophy [7], as well as accelerated collagen I and III synthesis by cardiac fibroblasts exposed to high glucose levels [8]. Increased plasma glucose levels also augment the generation of advanced glycation end-products (AGEs), the molecules formed by a nonenzymatic reaction between a reducing sugar and an amine group of proteins or lipids. AGEs stimulate the expression of extracellular matrix genes [38], and the tissue accumulation of AGEs has been shown to be associated with inappropriate LV mass increment [39]. In our study, T2DM patients, as compared to healthy volunteers of similar age, had significantly higher prevalence of inappropriate LV mass (57 vs 18%), i.e. LV mass higher than that necessary to sustain the body size and cardiac workload [33]. In fact, in healthy volunteers LV mass reflected the physiologic adaptation to body height and stroke work (a product of stroke volume and afterload), whereas in T2DM patients LV mass was determined also by HbA1c and T2DM duration (Table 4). Higher resting tension of hypertrophied cardiomyocytes together with interstitial fibrosis and collagen cross-linking [40] may explain the negative impact of LV mass increment on LV diastolic function.

The association between glycemic control and aortic stiffness is supposed to reflect AGEs-related collagen cross-linking within the arterial wall [41]. Stiffening of the aortic wall results in unfavorable alterations in central hemodynamics, that include an augmentation in forward arterial pressure wave amplitude and premature wave reflection, both of which increase central systolic BP; i.e. LV afterload [42]. A direct effect of afterload on LV relaxation has been demonstrated in experimental studies showing that afterload elevation slows down the LV pressure fall during isovolumic relaxation, leading to an incomplete myocardial lengthening [13, 14]. The rate of LV pressure fall slows in direct proportion to the magnitude of systolic pressure increment [14]. In our study, carotid systolic BP was inversely and independently associated with e′ velocity, both in healthy volunteers and in T2DM patients, and its magnitude was higher in diabetic population.

In contrast to aortic stiffness, which was negatively influenced by fasting plasma glucose in healthy volunteers and by HbA1c in T2DM patients, the local carotid stiffness was not independently related to metabolic parameters. This finding is consistent with results of previous studies demonstrating a different impact of modifiable cardiovascular risk factors on aortic and carotid stiffness [43, 44].

It should be emphasized that other T2DM-related factors, not evaluated in this study, like insulin-resistance and altered myocardial metabolism [45, 46], myocardial steatosis [47] or autonomic dysfunction [48], might participate on the impairment of LV diastolic performance in T2DM. In fact, in our T2DM patients, sex, age, carotid BP, heart rate and LV mass explained only 32% of e′ velocity variance.

### Systo-diastolic coupling and LV systolic performance

Early diastolic longitudinal velocity e′ is determined not only by LV relaxation rate but also by restoring forces [15, 16] generated during systolic contraction of myocardial fibers. Study in anesthetized dogs has suggested that the magnitude of restoring forces is matched to the force of LV contraction, and that the determinants of systolic shortening are also the determinants of e′ velocity [16]. In healthy volunteers of our study, s′ and e′ velocities closely correlated each other independently of other covariates (Table 4), whereas in T2DM patients the association between systolic and diastolic velocity was weaker (Fig. 1) and s′ velocity did not result an independent determinant of e′. Furthermore, T2DM patients, though having LV EF comparable to that of healthy volunteers, showed lower longitudinal systolic velocity s′ and ess-MWS. Altogether these findings imply that myocardial metabolic and structural changes related to diabetes affect systo-diastolic coupling and provoke a subclinical decline in systolic myocardial performance, both longitudinal and circumferential [34].

### Glycemic control

Asymptomatic T2DM patients with sub-optimal glycemic control (Hb1Ac ≥ 6.5%) [27] had significantly lower diastolic and systolic longitudinal velocities and ess-MWS together with higher aortic stiffness, central BP and LV mass index when compared to those with HbA1c <6.5%. These observations are in agreement with prospective studies demonstrating that an improvement in glycemic control is paralleled by improvements in LV systolic and diastolic function [21, 49].

### Study limitations

The cross-sectional design of the study cannot elucidate cause-and-effect relationships. Silent inducible ischemia was not assessed by stress testing in T2DM patients. Data on other T2DM-related factors that might influence LV mass and arterial stiffness, like plasma insulin and adiponectin, were not available. In healthy volunteers, HbA1c was not determined. LV longitudinal function was estimated by a simple measure of mitral annulus velocity and not by a more complex measurement of LV strain, since speckle tracking–based strain imaging has low achievable frame rates [15], and since previous studies have demonstrated a good association of e′ velocity with LV relaxation rate and restoring forces [15, 16].

## Conclusions

Chronic increase in plasma glucose level in T2DM patients is associated with aortic stiffening and consequent increase in LV afterload, as well as with LV mass increment. Increase in both LV afterload and mass showed a negative impact on e′ longitudinal myocardial velocity used as a marker of LV diastolic function. LV systo-diastolic coupling and systolic myocardial performance seem to be also negatively influenced by diabetes and increased plasma glucose, even if the chamber function is still preserved. An adequate control of plasma glucose level and central BP might prevent the development of LV myocardial dysfunction and its eventual transition towards heart failure.

## Abbreviations

LV: left ventricle
T2DM: type 2 diabetes mellitus
cfPWV: carotid-femoral pulse wave velocity
ccaPWV: carotid pulse wave velocity
BMI: body mass index
HbA1c: glycosylated hemoglobin
AGEs: advanced glycation end-products
